# Assessment and decomposition of excessive red meat consumption among hypertensive adults: A Wagstaff decomposition analysis

**DOI:** 10.1371/journal.pone.0359650

**Published:** 2026-10-05

**Authors:** Saba Karimi, Sara Kaveh, Sara Emamgholipour sefiddashti, Samaneh Akbarpour, Sajjad Dorri, Yosra Azizpour

**Affiliations:** 1 Non-Communicable Diseases Research Center, Endocrinology and Metabolism Population Sciences Institute, Tehran University of Medical Sciences, Tehran, Iran; 2 Students' Scientific Research Center, Tehran University of Medical Sciences, Tehran, Iran; 3 Department of Health Management, Policy and Economics, School of Public Health, Tehran University of Medical Sciences, Tehran, Iran; 4 Sleep Breathing Disorders Research Center (SBDRC), Tehran University of Medical Sciences, Tehran, Iran; PLOS: Public Library of Science, UNITED KINGDOM OF GREAT BRITAIN AND NORTHERN IRELAND

## Abstract

**Introduction:**

Red meat consumption is an important dietary factor in hypertension management, yet its distribution may vary across socioeconomic groups. This study examines wealth-related inequality in red meat consumption among hypertensive adults in Iran.

**Objective:**

To quantify wealth-related inequality in red meat consumption among hypertensive adults in Iran and explore factors associated with this inequality.

**Methods:**

Utilizing data from the 2021 Iranian STEPS survey, a nationally representative sample of 9,024 hypertensive adults was analyzed. Excessive consumption was defined as consuming red meat four or more times per week. Wealth-related inequality in consumption was then assessed using a wealth index derived from principal component analysis (PCA) of household assets and living conditions. Wagstaff decomposition analysis was employed to disaggregate the concentration index (CI) and explore the contribution of each factor to the observed inequality.

**Results:**

The hypertensive adults included in the study had a mean age (95% CI) of 56.1 years (55.8, 56.5). The proportion of excessive red meat consumption was 16.5%. A significant wealth-related inequality was observed in excessive red meat consumption (CI = 0.179, p  <  0.001), indicating that excessive consumption was concentrated among wealthier individuals. Wagstaff decomposition analysis showed that higher educational attainment (≥12 years) was the largest contributor to the observed inequality (20.6%), followed by unpaid work (6.8%), unemployment (4.4%), and age 40–59 years (4.2%). Overall, the included determinants explained 29.4% of the observed inequality.

**Conclusion:**

This study identified significant wealth-related inequality in excessive red meat consumption among hypertensive adults in Iran, with higher consumption concentrated among wealthier groups. Educational attainment was the largest contributor to the observed inequality. Policies should consider socioeconomic differences in dietary behaviors and promote targeted nutrition interventions to reduce cardiovascular risk and improve dietary equity.

## Introduction

Hypertension is one of the leading global health challenges, affecting about one in three adults aged 30–79 years and contributing substantially to cardiovascular disease (CVD), stroke, and premature mortality [1,2]. Its prevalence remains relatively consistent across countries, ranging only slightly, from 32% in high-income settings to 34% in low-income settings [2]. Emerging evidence further indicates that excessive meat consumption, particularly of red and processed varieties, is a significant risk factor for hypertension [3,4]. Despite this burden, a considerable proportion of individuals with hypertension remain undiagnosed, and among those diagnosed, many do not receive treatment or fail to achieve adequate blood pressure control [1,2].

Dietary modification is a cornerstone of hypertension management [5]. Simulation studies suggest that replacing half of meat and dairy with plant-based foods could prevent approximately 37000 deaths [6]. Cohort analyses further indicate that even relatively low levels of red and processed meat consumption are positively associated with both all-cause and cardiovascular mortality when compared with zero intake [7]. Despite such evidence, a substantial proportion of adults with hypertension still do not meet dietary recommendations, reflecting excessiveor unhealthy patterns of red meat consumption that may worsen disease progression and elevate the risk of cardiovascular complications [8].

Importantly, dietary behaviors are not uniformly distributed across populations. Socioeconomic and demographic factors—including income, education, employment status, and place of residence—play a critical role in shaping access to and preferences for different food types [9]. As a result, inequalities in red meat consumption patterns may be associated with wider disparities in hypertension outcomes, particularly in resource-constrained settings [10]. However, there is a notable scarcity of equity-focused research in low- and middle-income countries (LMICs), including Iran. Most existing studies emphasize overall consumption levels or health associations, without specifically examining the socioeconomic determinants of excessive red meat intake among high-risk subgroups such as hypertensive adults. Understanding these inequities is essential for guiding evidence-based public health strategies that can improve dietary quality and promote equity in health outcomes.

To address this challenge, analytical methods capable of disentangling the sources of inequality are required. Wagstaff’s decomposition analysis, widely applied in health economics to quantify wealth-related inequalities, helps identify the relative contribution of key determinants [11]. This study is among the first to apply Wagstaff decomposition to red meat consumption patterns specifically in a hypertensive population, moving beyond descriptive estimates to quantify the determinants of wealth-related inequality. This study seeks to quantify the wealth-related inequality in excessive red meat consumption among hypertensive adults in Iran and decompose its contributing factors using Wagstaff analysis.

## Methods

### Data source and study population

The present analysis was based on data from the STEPwise Approach to non-communicable disease (NCD)Risk Factor Surveillance (STEPS) survey in 2021 in Iran, a nationwide cross-sectional study designed to monitor major NCD risk factors. Participant recruitment for this study started in early 2020, was temporarily halted due to COVID-19, and resumed in early 2021 and continued until completion. A representative sample of adults aged 18 years and above was selected using a systematic cluster random sampling approach. Sampling was carried out across all 31 provinces, with proportional allocation based on provincial population size. In total, 3,176 clusters were defined, each including nine individuals from both urban and rural settings, yielding a final sample of 27,874 adults [12]. Individuals were eligible if they were Iranian residents at the time of the survey and met the minimum age requirement. Participants with conditions that prevented meaningful participation, those unable to provide biological samples, or pregnant women were excluded. Prior to enrollment, informed consent was obtained, and participants were assured of confidentiality and data protection [12].

In the present study, a subpopulation of individuals with hypertension was selected. Hypertension was defined as systolic blood pressure (SBP) of 140 mmHg or higher, diastolic blood pressure (DBP) of 90 mmHg or higher, or through self-reported, physician diagnosis or current use of antihypertensive medications. Of 9,572 hypertensive individuals, 548 were excluded due to incomplete information on key variables, including meat consumption (n = 10) wealth index (n = 459), education level (n = 58), comorbidity (n = 21). Observations with missing values were excluded using complete-case analysis. The final analytic sample consisted of 9,024 participants (Fig 1).

**Fig 1:**
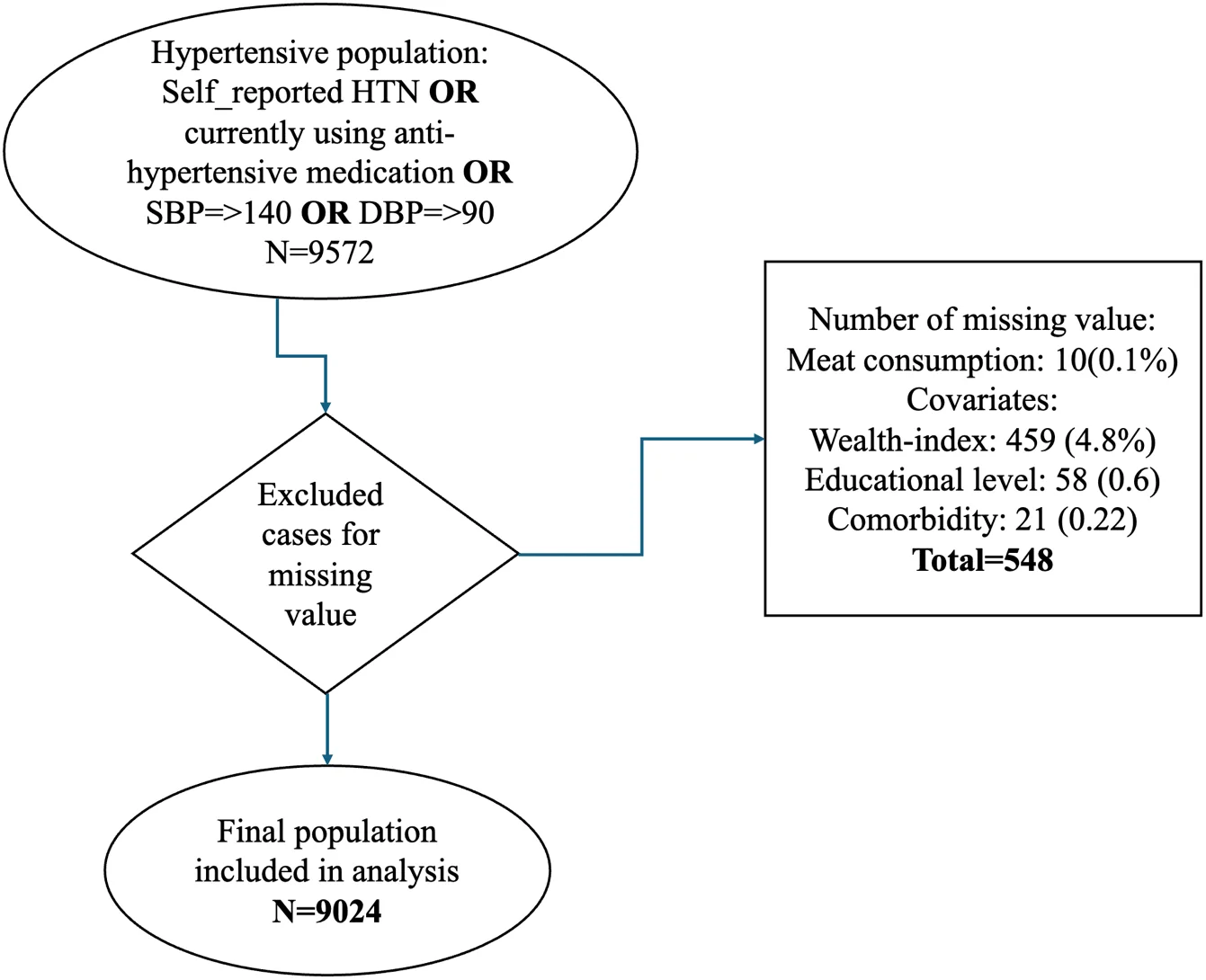
Flowchart of the study population.

### Definition of variables

#### Outcome variable.

The outcome variable was excessive red meat consumption. In the STEPS survey, participants were asked: “How often do you usually consume red meat such as beef, veal, lamb, goat, mutton, or camel?” Response options included: (1) less than once a month or never, (2) one to three times per month, (3) one to three times per week, (4) four to six times per week, (5) one to two times per day, and (6) three or more times per day. Participants reporting red meat consumption four or more times per week were classified as excessive consumers. This threshold was selected to identify frequent red meat consumption, consistent with evidence suggesting that higher levels of red meat intake are associated with adverse health outcomes [13–15].

#### Covariates.

Age was calculated based on the date of birth recorded in the questionnaire and categorized into three groups: 18–39 years, 40–59 years, and 60 years or older. Sex was self-reported and classified as male or female. Marital status was categorized into two groups: married and single, with the single category including never-married, divorced, and widowed participants. Area of residence was determined based on the address recorded in the questionnaire and classified into two categories: urban and rural. Education level was categorized into four groups: no formal education, 1–6 years of schooling, 7–11 years, and 12 years or more. Job status was classified as employed, unemployed, unpaid work, or retired.

Comorbidity was defined as the presence of at least one additional chronic condition among individuals with hypertension. Four chronic conditions were considered: diabetes, hypercholesterolemia, CVD, and cancer. Diabetes, hypercholesterolemia, and CVD were defined as self-reported conditions if participants indicated that a physician or health worker had ever diagnosed them with the respective condition, or if they were currently taking medication for it. Cancer was defined as a self-reported condition if participants reported that, within the past 12 years, a physician or health worker had told them that they had cancer. Each condition was coded as a binary variable (yes/no). A comorbidity indicator was then created by summing these variables. Participants were categorized into two groups: no comorbidity (no additional chronic condition) and any comorbidity (presence of at least one of the specified chronic conditions in addition to hypertension).

#### Wealth index.

The wealth index was constructed through principal component analysis (PCA) of household assets and housing characteristics, including car ownership, housing quality indicators (such as floor and wall materials), access to basic utilities, and selected durable goods. The derived wealth score was subsequently categorized into five quintiles, from the poorest to the wealthiest.

### Statistical analysis

Inequality in red meat consumption among individuals with hypertension was estimated using survey sample weights to ensure national representativeness. Weighting procedures were implemented after data cleaning and included adjustments for overall and stage-specific non-response (with age-targeted corrections considering the impact of the COVID-19 pandemic), followed by post-stratification by province, residential area, sex, and age [12]. All weights were subsequently combined and applied in the final analyses to reduce potential bias and improve the generalizability of results. Descriptive statistics were presented using percentages and 95% confidence intervals (CIs).

We first calculated inequality in excessive red meat consumption using the concentration curve (CC) and the concentration index (CI), which are two widely recognized and commonly used methods for assessing wealth-related inequalities across socioeconomic groups [11,16]. The CC offers a visual representation of inequality by plotting the cumulative proportion of the outcome variable (y-axis) against the cumulative proportion of the population ranked from poorest to richest (x-axis). A 45-degree line represents perfect equality. If the curve lies above this line, the outcome is concentrated among poorer individuals (pro-poor inequality), while a curve below the line indicates concentration among wealthier groups (pro-rich inequality) [17]. The further the curve is from the equality line, the greater the inequality.

The CI is twice the area between the concentration curve and the line of equality and provides a concise numerical summary of the CC [18]. It is calculated as twice the covariance between the outcome variable and the fractional rank of individuals in the socioeconomic distribution, divided by the mean of the outcome. It can be expressed as (equation 1) [19]:


$$\begin{array}{c} \text{CI}=\frac{\text{2}}{\mu }.\text{cov }({\text{y}}_{\text{i}},{\text{R}}_{\text{i}}) \end{array}$$ (1)

Here, y_i_ is the outcome for individual i, μ is its mean or the proportion of the health variable, and R_i_ represents the fractional rank in the wealth distribution [19]. A positive CI means the outcome is more common among the richer population, a negative CI indicates concentration among the poorer, and a value of zero represents perfect equality.

For binary outcomes, the theoretical limits of the CI are not fixed at –1 and +1 but instead depend on the mean of the variable [20]. To make the results comparable and interpretable, we used the Wagstaff normalization, which can be expressed as (equation 2) [20]:


$$\begin{array}{c} {\text{CI}}_{\text{Wagstaff}}\;=\frac{CI}{\text{1}-\mu } \end{array}$$ (2)

Where μ represents the mean of the outcome, and CI refers to the standard concentration index. This adjustment ensures that the index remains within the –1 to +1 range, providing a clear and consistent framework for interpreting pro-poor and pro-rich inequalities.

Second, we applied the Wagstaff decomposition approach [11] to decompose the CI and determine how much each explanatory variable contributes to the overall inequality [17,21]. This technique is based on regression analysis of the association between the outcome variable and its determinants for any linear additive regression model of health outcome, such as (equation 3):


$$\begin{array}{c} \text{y}=\alpha +\sum \text{k }\beta_{\text{k}}{\text{x}}_{k}^{-}+\epsilon \end{array}$$ (3)

The CI for y can be written as follows [11,17] (equation 4):


$$\begin{array}{c} \text{CI}=\sum \text{k }(\frac{\beta_{k}\;{\text{x}}_{k}^{-}}{\mu_{k}}\;).{\text{CI}}_{\text{k}}\;+\;\frac{\text{GC}\epsilon }{\mu } \end{array}$$ (4)

where µ_k_ represents the mean of y, xˉ_k_ is the mean of the x_k_, CI_k_ is the concentration index of x_k_, and GCε denotes the generalized concentration index of ε. Equation (4) indicates that the overall CI can be expressed as a weighted sum of the concentration indices of the k regressors, with the weight for each x_k_ given by the elasticity of y with respect to x_k_ (η_k_ = β_k_$\frac{{\text{x}}^{-}\text{k}}{\mu }$). The residual component represents the proportion of inequality unexplained by the included variables.

Because the outcome variable (excessive red meat consumption) was binary, a logistic regression model was fitted. Average marginal effects (dy/dx) were estimated and used instead of the log-odds coefficients in the decomposition analysis to facilitate interpretation [17]. This linear approximation is widely accepted and provides a clear picture of how each factor contributes to inequality [22]. Equation (5) shows the linear approximation used for the non-linear estimates:


$$\begin{array}{c} \text{CI}=\sum \text{k }(\frac{{\beta^{m}}_{k}\;{\text{x}}_{k}^{-}}{\mu_{k}}\;).\text{CIk }+\;\frac{\text{GC}\epsilon }{\mu } \end{array}$$ (5)

Where ${\beta^{m}}_{k}\;$ is the marginal effect (dy/dx) of each x. Finally, the percentage contribution of each determinant was derived using the adjusted method proposed by Yiengprugsawan et al. [23], which accounts for the total explained component aligned with the direction of the overall CI, offering a more accurate interpretation of the contributions.

Multicollinearity among explanatory variables was assessed using variance inflation factors (VIF), and no evidence of problematic collinearity was observed (maximum VIF = 2.23).

All statistical analyses were conducted using Stata version 17 (StataCorp, College Station, TX, USA), with the conindex command used to calculate the CI.

### Ethics statement

Ethical approval for this study was obtained from the Research Ethics Committee of the Endocrinology & Metabolism Research Institute at Tehran University of Medical Sciences (Approval No. IR.TUMS.EMRI.REC.1403.111) on 25 November 2024. The data used in this study were derived from the original study, in which all participants were aged 18 years or older, provided written informed consent, and all procedures were conducted in accordance with the principles of the Declaration of Helsinki.

## Results

Table 1 provides an overview of the baseline characteristics of the study population. The study comprised 9,024 adults aged 18 years and older, with a mean age of 56.1 [95% CI: 55.8, 56.5] years. The proportion of excessive red meat consumption was 16.5%. The distribution of red meat consumption among participants reveals demographic variations.

**Table 1:** Distribution of the baseline characteristics by red meat consumption (Non-Excessive vs. Excessive).

Variables	Categories	n	Total %	Non-Excessive %	Excessive %	p-value
Age	18–39	1,231	13.16 [12.41, 13.96]	13.3 [12.48, 14.18]	12.46 [10.72, 14.43]	0.02
	40–59	3,904	43.53 [42.38, 44.68]	42.8 [41.55, 44.07]	47.18 [44.38, 50]	
	≥60	3,889	43.31 [42.17, 44.46]	43.89 [42.64, 45.16]	40.36 [37.64, 43.15]	
Sex	Female	5,010	55.53 [54.38, 56.68]	55.86 [54.6, 57.12]	53.87 [51.05, 56.66]	0.20
	Male	4,014	44.47 [43.32, 45.62]	44.14 [42.88, 45.4]	46.13 [43.34, 48.95]	
Area of Residence	Urban	6,626	75.22 [74.23, 76.19]	74.88 [73.78, 75.94]	76.94 [74.54, 79.19]	0.12
	Rural	2,398	24.78 [23.81, 25.77]	25.12 [24.06, 26.22]	23.06 [20.81, 25.46]	
Marital Status	Married	7,273	80.41 [79.47, 81.3]	80 [78.97, 80.99]	82.45 [80.17, 84.51]	0.053
	Single	1,751	19.59 [18.69, 20.53]	20 [19.01, 21.03]	17.55 [15.49, 19.83]	
Years of Schooling	0	2387	24.97 [24, 25.97]	26.18 [25.09, 27.29]	18.88 [16.84, 21.1]	<0.001
	1–6	2,802	30.7 [29.65, 31.78]	30.6 [29.44, 31.77]	31.24 [28.68, 33.92]	
	7–11	1,368	15.77 [14.94, 16.64]	15.75 [14.84, 16.71]	15.88 [13.92, 18.07]	
	≥12	2,467	28.55 [27.51, 29.62]	27.48 [26.34, 28.64]	34 [31.37, 36.73]	
Job Status	Employed	2,497	27.98 [26.95, 29.03]	27.25 [26.13, 28.4]	31.66 [29.09, 34.34]	<0.001
	Unpaid work	4,565	50.39 [49.24, 51.55]	50.96 [49.69, 52.23]	47.53 [44.73, 50.35]	
	Unemployed	479	4.93 [4.47, 5.44]	5.4 [4.86, 5.98]	2.59 [1.85, 3.60]	
	Retired	1,483	16.70 [15.84, 17.58]	16.39 [15.46, 17.36]	18.23 [16.17, 20.49]	
Comorbidity	No	4,761	52.34 [51.18, 53.49]	52.06 [50.79, 53.33]	53.73 [50.91, 56.52]	0.29
	Yes	4,263	47.66 [46.51, 48.82]	47.94 [46.67, 49.21]	46.27 [43.48, 49.09]	
Wealth Quintile	Poorest	2,060	21.47 [20.55, 22.42]	23.34 [22.3, 24.42]	12.04 [10.39, 13.91]	<0.001
	Poor	1,891	21.63 [20.69, 22.61]	21.9 [20.87, 22.98]	20.26 [18.08, 22.63]	
	Middle	1,812	19.52 [18.63, 20.45]	20.19 [19.19, 21.22]	16.18 [14.24, 18.32]	
	Rich	1,739	19.24 [18.34, 20.17]	18.49 [17.52, 19.5]	23.04 [20.74, 25.51]	
	Richest	1,522	18.13 [17.24, 19.05]	16.08 [15.15, 17.06]	28.48 [25.98, 31.13]	

**Footnote:** Percentages were weighted to account for the survey design, and the values are reported as weighted % with 95% confidence intervals.

Excessive red meat consumption was defined as consuming red meat four or more times per week.

Percentages may not total 100% due to rounding.

The distribution of age differed significantly between the non-excessive and excessive red meat consumption groups (p-value = 0.02). Compared with the non-excessive consumption group, the excessive consumption group included a higher proportion of participants aged 40–59 years (47.18% vs. 42.8%) and lower proportions of participants aged 18–39 years (12.46% vs. 13.30%) and ≥60 years (40.36% vs. 43.89%). In contrast, the distribution of sex was similar between the two groups (p-value = 0.20), although the excessive consumption group had a slightly lower proportion of females (53.87% vs. 55.86%) and a slightly higher proportion of males (46.13% vs. 44.14%). Likewise, no significant difference was observed in the distribution of area of residence (p-value = 0.12); however, the excessive consumption group had a marginally higher proportion of urban residents (76.94% vs. 74.88%) and a lower proportion of rural residents (23.06% vs. 25.12%) than the non-excessive consumption group.

The Wagstaff normalized CI revealed a significant wealth-related inequality in excessive red meat consumption (CI = 0.179, p <  0.001), indicating that excessive consumption was disproportionately concentrated among wealthier individuals. As shown in Fig 2, the proportion of excessive consumers rose from 12.04% (95% CI: 10.39, 13.91) in the poorest quintile to 28.48% (25.98, 31.13) in the richest quintile. This pattern demonstrates a clear wealth gradient in excessive red meat consumption among hypertensive adults.

**Fig 2:**
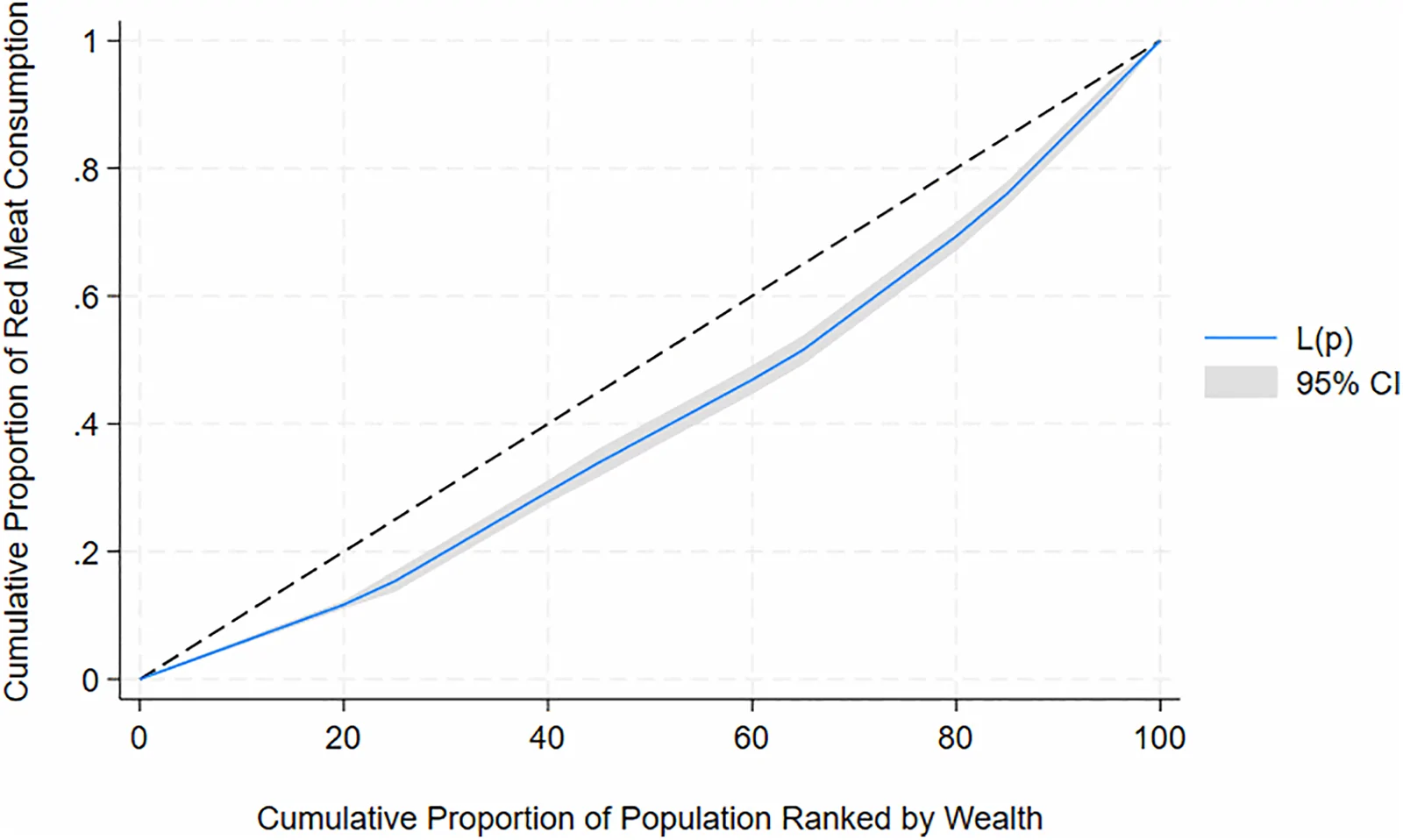
Concentration curve for the wealth-related inequality in excessive red meat consumption.

Wagstaff decomposition analysis was conducted to quantify the contribution of different determinants to wealth-related inequality in excessive red meat consumption. The included variables explained 29.41% of the observed inequality, while 70.59% remained unexplained. Educational attainment was the largest contributor, with individuals having ≥12 years of schooling contributing positively to inequality and accounting for 20.64% of the total concentration index, indicating that higher education was disproportionately concentrated among wealthier individuals and associated with higher probability of excessive red meat consumption. Employment status also played a significant role, where unpaid work and unemployment contributed positively to inequality (6.76% and 4.38%, respectively), thereby increasing the observed pro-rich inequality (Table 2).

Among demographic characteristics, individuals aged 40–59 years contributed positively to inequality (4.21%), indicating an inequality-increasing effect, whereas age ≥ 60 years (−4.71%) and male sex (−3.45%) contributed negatively to the overall CI, suggesting a mitigating effect on wealth-related inequality. Being married increased inequality (2.46%), while urban residence had a negligible positive contribution (0.21%). Overall, the results indicate that higher educational attainment and adverse employment conditions were the primary drivers of wealth-related inequality in excessive red meat consumption, whereas older age and male sex partially offset this inequality (Table 2).

**Table 2:** Wagstaff decomposition analysis of wealth-related inequality in excessive red meat consumption.

Variables	Categories	Marginal Effect	Means	Elasticity	CI of variable	Absolute Contribution	Contribution %
Age	18–39 (ref)	–	–	–	–	–	–
	40–59	0.034*	0.435	0.090	0.084	0.008	4.206
	≥60	0.030*	0.433	0.079	−0.107	−0.008	−4.706
Sex	Female (ref)	–	–	–	–	–	–
	Male	−0.029***	0.445	−0.078	0.079	−0.006	−3.452
Area of Residents	Rural (ref)	–	–	–	–	–	–
	Urban	0.001**	0.752	0.005	0.082	0.000	0.209
Marital Status	Single (ref)	–	–	–	–	–	–
	Married	0.018**	0.806	0.088	0.050	0.004	2.456
Years of Schooling	0 (ref)	–	–	–	–	–	–
	1–6	0.024**	0.311	0.045	−0.085	−0.004	−2.148
	7–11	0.033*	0.158	0.032	0.096	0.003	1.695
	≥12	0.065*	0.286	0.113	0.328	0.037	20.645
Job Status	Employed (ref)	–	–	–	–	–	–
	Unpaid work	−0.04***	0.504	−0.122	−0.099	0.012	6.758
	Unemployed	−0.08**	0.059	−0.029	−0.274	0.008	4.379
	Retired	−0.006**	0.167	−0.006	0.191	−0.001	−0.648
Comorbidity	No (ref)	–	–	–	–	–	–
	Yes	−0.004**	0.472	−0.011	0.000	0.000	0.000
Total	–	–	–	–	–	0.053	29.41%
CI_y_	_	_	_	_	_	0.179	100
Residual	–	–	–	–	–	0.126	70.59%

**Footnote:** The table presents results from the Wagstaff decomposition of the CI for excessive red meat consumption. Positive percentage contributions indicate that the variable increases inequality, whereas negative values imply a reduction in inequality. The sum of contributions approximates the overall observed inequality (CI = 0.179, p  <  0.001). Abbreviations: CI, Concentration Index; Ref, Reference Category.

Statistical significance of marginal effects is indicated as follows: *p < 0.1, **p < 0.05, ***p < 0.01

## Discussion

This study provides evidence on wealth-related inequality in red meat consumption among hypertensive adults in Iran, revealing that excessive intake is disproportionately concentrated among wealthier individuals. This finding is consistent with evidence from several settings showing that higher socioeconomic position is often associated with greater consumption of animal-source foods, including red meat, because of increased purchasing power and affordability [9,24,25]. These economic factors may be further influenced by broader macroeconomic pressures. Previous research suggests that international trade restrictions have been associated with reduced purchasing power and rising food prices [26]. This economic context may contribute to the observed disparities examined in our study.

Educational attainment emerged as the strongest contributor to the observed dietary inequality. This finding may reflect the role of education in shaping dietary behaviors through multiple pathways, including socioeconomic opportunities and attitudinal factors related to food choices. However, evidence suggests that the relationship between education and meat consumption is context-dependent and may be influenced by broader socioeconomic structures and climate-related attitudes [27,28]. Therefore, the substantial unexplained component observed in our decomposition may partly reflect unmeasured factors, such as environmental concerns, preferences, and nutritional beliefs, which were not captured in the current dataset.

Individuals with ≥12 years of schooling accounted for approximately 20.6% of the observed inequality, making education the largest contributor to the pro-rich concentration of excessive red meat consumption. Higher educational attainment was disproportionately concentrated among wealthier individuals and therefore contributed substantially to the observed socioeconomic inequality. Although higher education is often associated with healthier dietary behaviors and greater nutritional awareness [29–31], the relationship between education and specific food choices may vary across cultural and economic contexts. In settings where red meat is relatively expensive, individuals with higher educational attainment and greater economic resources may have more opportunities to consume red meat frequently despite awareness of dietary recommendations. Conversely, the negative contribution observed for some lower educational categories suggests that less-educated individuals were less likely to participate in the pro-rich pattern of excessive consumption. Given the relatively high cost of red meat in Iran, this finding may partly reflect economic constraints that limit frequent consumption among socioeconomically disadvantaged groups. Taken together, these findings suggest that educational attainment influences meat consumption through complex socioeconomic and behavioral pathways rather than through education alone [27,28,32,33].

Area of residence (urban vs rural) contributed only minimally to the observed inequality (0.21%), suggesting that differences in excessive red meat consumption were driven primarily by socioeconomic characteristics rather than residential location. Nevertheless, previous studies have shown that rural–urban differences in food availability, affordability, and dietary culture can influence meat consumption patterns [34]. The limited contribution observed in our study may indicate that wealth-related differences outweigh geographic disparities among hypertensive adults in Iran.

Age and life stage were associated with inequality patterns. Adults aged 40–59 years contributed positively to inequality (4.2%), whereas those aged ≥60 years contributed negatively (−4.7%), indicating that older age partially offset the observed pro-rich concentration of excessive red meat consumption. Genders Sex differences were also evident. Male sex reduced the observed inequality (−3.5%), suggesting that men were more evenly distributed across wealth groups with respect to excessive consumption. Previous studies have reported substantial variation in meat consumption according to age and sex, although the direction and magnitude of these associations differ across settings [35–38]. Such findings suggest that demographic characteristics interact with socioeconomic conditions in shaping dietary behavior.

Marital status and employment characteristics also contributed to the observed inequality. Married individuals increased the pro-rich inequality by approximately 2.5%, while unpaid work and unemployment contributed 6.8% and 4.4%, respectively. These findings suggest that labor market position and household structure may influence dietary behavior through their effects on economic resources, food purchasing decisions, and access to dietary choices. Retirement had a small inequality-reducing effect.

Comorbidity showed a negligible contribution to the observed inequality in excessive red meat consumption. Although individuals with multiple chronic conditions may experience greater healthcare needs and are often expected to receive more dietary counseling, the presence of additional chronic diseases did not substantially explain socioeconomic differences in excessive red meat intake in our study. This finding suggests that, among hypertensive adults, dietary inequalities may be driven more strongly by broader social determinants, including education, economic resources, and food environments, rather than by clinical characteristics alone. Previous evidence indicates that multimorbidity is closely linked with socioeconomic disadvantage and healthcare burden; however, the relationship between multimorbidity and specific dietary behaviors may depend on the social and cultural context [39].

A substantial portion of the wealth-related inequality in excessive red meat consumption remained unexplained by the variables included in the decomposition model. Specifically, the measured determinants accounted for only 29.4% of the observed concentration index, while approximately 70.59% remained unexplained. Although the explained component was consistent with the direction of the observed concentration index, the magnitude of the residual component indicates that the included variables captured only a limited proportion of the mechanisms underlying wealth-related inequality. The large residual suggests that important determinants of excessive red meat consumption were not measured in the STEPS survey. Potential contributors include food prices, household food expenditure, dietary preferences, cultural norms, nutrition knowledge, local food environments, and intra-household allocation of food resources. These factors, highlighted in the broader literature on food equity and consumer behavior [40–42], indicate that dietary disparities are embedded in deeper social and cultural structures, including food preferences, consumption norms, and household dietary practices, beyond direct economic conditions alone. Future studies incorporating these factors may provide a more comprehensive understanding of the mechanisms driving socioeconomic inequality in dietary behavior.

The findings suggest that socioeconomic disparities in excessive red meat consumption are shaped by systemic factors, including educational attainment, employment status, and broader socioeconomic conditions, rather than individual choice alone. These results highlight the need for targeted policy responses that address the social and behavioral determinants of dietary patterns among hypertensive adults. Potential interventions may include strengthening nutrition counseling within hypertension management programs, improving awareness of the cardiovascular risks associated with excessive red meat consumption, and promoting balanced dietary patterns consistent with national dietary guidelines. Such strategies should be tailored to different socioeconomic groups, recognizing that both economic resources and social characteristics influence dietary choices. Implementing these approaches through Iran’s primary healthcare system and fostering cross-sectoral collaboration may help reduce diet-related inequalities and improve cardiovascular health outcomes.

Our findings should be interpreted in light of several limitations. The cross-sectional designlimits causal inference regarding the association between wealth and consumption. Furthermore, reliance on self-reported dietary data introduces potential recall bias, and the absence of details on processed meat intake, cooking methods, and portion sizes precludes a more complete nutritional assessment. Another important limitation relates to data accessibility. Although the STEPS survey provides a nationally representative dataset, access to the underlying individual-level data requires formal institutional approval and is not publicly available. As a result, independent replication and external verification of the decomposition analyses may be challenging, which may limit the transparency and reproducibility of the findings. Finally, the wealth index, while informative, is a static measure that may not reflect short-term income fluctuations or intra-household financial dynamics, which are crucial determinants of actual food access.

## Conclusion

Overall, this study highlights significant wealth-related disparities in in excessive red meat consumption among hypertensive adults in Iran and identifies key social factors associated with these inequalities. Excessive consumption was concentrated among wealthier individuals, with educational attainment representing the largest contributor to the observed inequality. These findings suggest that socioeconomic advantage may facilitate greater exposure to dietary behaviors associated with cardiovascular risk. By applying concentration indices and Wagstaff decomposition, this study provides evidence that can support more targeted and equitable dietary interventions for hypertensive populations.

## Abbreviations

CC: Concentration Curve
CI: Concentration Index
CIs: Confidence Intervals
DBP: Diastolic blood pressure
NCDs: Non-communicable diseases
PCA: Principal component analysis
SBP: Systolic blood pressure
STEPS: STEPwise approach to NCD risk factor surveillance
WHO: World Health Organization;
CVD: Cardiovascular disease;
LMICs,: Low- and middle-income countries;
VIF: Variance inflation factor;
COVID-19,: Coronavirus disease 2019.

## References

[1] Kario K, Okura A, Hoshide S, Mogi M. The WHO Global report 2023 on hypertension warning the emerging hypertension burden in globe and its treatment strategy. Hypertens Res. 2024;47(5):1099–102. doi: 10.1038/s41440-024-01622-w 38443614

[2] World Health Organization. Global report on hypertension: The race against a silent killer;Geneva: WHO; 2023.

[3] Allen TS, Najem M, Wood AC, Lee DJ, Pacheco LS, Daniels LB, et al. Red meat consumption and hypertension: An updated review. Curr Cardiol Rep. 2025;27(1):50. 10.1007/s11886-025-02201-2 PMC11811473 39928063

[4] Zhuang P, Jiao J, Wu F, Mao L, Zhang Y. Associations of meat consumption and changes with all-cause mortality in hypertensive patients during 11.4-year follow-up: Findings from a population-based nationwide cohort. Clin Nutr. 2021;40(3):1077–84. doi: 10.1016/j.clnu.2020.06.040 32741682

[5] Altawili AA, Altawili M, Alwadai AM, Alahmadi AS, Alshehri AMA, Muyini BH, et al. An exploration of dietary strategies for hypertension management: A narrative review. Cureus. 2023;15(12):e50130. doi: 10.7759/cureus.50130 38186513 PMC10771610

[6] Scarborough P, Allender S, Clarke D, Wickramasinghe K, Rayner M. Modelling the health impact of environmentally Sustainable dietary scenarios in the UK. Eur J Clin Nutr. 2012;66(6):710–5. doi: 10.1038/ejcn.2012.34 22491494 PMC3389618

[7] Alshahrani SM, Fraser GE, Sabaté J, Knutsen R, Shavlik D, Mashchak A, et al. Red and processed meat and mortality in a low meat intake population. Nutrients. 2019;11(3):622. doi: 10.3390/nu11030622 30875776 PMC6470727

[8] Shim JS, Oh K, Jung SJ, Kim HC. Self-reported diet management and adherence to dietary guidelines in Korean adults with hypertension. Korean Circ J. 2020;50(5):432–40. doi: 10.4070/kcj.2019.0230 32096363 PMC7098818

[9] Clonan A, Roberts KE, Holdsworth M. Socioeconomic and demographic drivers of red and processed meat consumption: Implications for health and environmental sustainability. Proc Nutr Soc. 2016;75(3):367–73. doi: 10.1017/S0029665116000100 27021468 PMC4974628

[10] Ba DM, Gao X, Chinchilli VM, Liao D, Richie JP Jr, Al-Shaar L. Red and processed meat consumption and food insecurity are associated with hypertension; Analysis of the National Health and Nutrition Examination Survey data, 2003-2016. J Hypertens. 2022;40(3):553–60. doi: 10.1097/HJH.0000000000003048 34784309

[11] Wagstaff A, van Doorslaer E, Watanabe N. On decomposing the causes of health sector inequalities with an application to malnutrition inequalities in Vietnam. J Econom. 2003;112(1):207–23. doi: 10.1016/s0304-4076(02)00161-6

[12] Djalalinia S, Azadnajafabad S, Ghasemi E, Yoosefi M, Rezaei N, Farzi Y, et al. Protocol design for surveillance of risk factors of non-communicable diseases during the COVID-19 pandemic: An experience from Iran STEPS survey 2021. Arch Iran Med. 2022;25(9):634–46. doi: 10.34172/aim.2022.99 37543889 PMC10685773

[13] Diet, nutrition and the prevention of chronic diseases. Geneva: WHO/FAO; 2002.

[14] Godfray HCJ, Aveyard P, Garnett T, Hall JW, Key TJ, Lorimer J, et al. Meat consumption, health, and the environment. Science. 2018;361(6399):eaam5324. doi: 10.1126/science.aam5324 30026199

[15] Mahan LK. Krause’s food & the nutrition care process. 14th ed. St. Louis: Elsevier Health Sciences; 2016.

[16] Wagstaff A, Paci P, van Doorslaer E. On the measurement of inequalities in health. Social Science & Medicine. 2002;33(5):545–57. 10.1016/0277-9536(91)90212-U 1962226

[17] Wagstaff A, O’Donnell O, Van Doorslaer E, Lindelow M. Analyzing health equity using household survey data: A guide to techniques and their implementation. World Bank Publications; 2007.

[18] O’Donnell O, O’Neill S, Van Ourti T, Walsh B. conindex: Estimation of concentration indices. Stata J. 2016;16(1):112–38. doi: 10.1177/1536867x1601600112 27053927 PMC4819995

[19] Kakwani N, Wagstaff A, van Doorslaer E. Socioeconomic inequalities in health: Measurement, computation, and statistical inference. J Econom. 1997;77(1):87–103. doi: 10.1016/s0304-4076(96)01807-6

[20] Wagstaff A. The bounds of the concentration index when the variable of interest is binary, with an application to immunization inequality. Health Econ. 2005;14(4):429–32. doi: 10.1002/hec.953 15495147

[21] Wagstaff A. Socioeconomic inequalities in child mortality: comparisons across nine developing countries. Bull World Health Organ. 2000;78(1):19–29. 10686730; PMCID: PMC2560599. PMC2560599

[22] Zhao Y. Decomposition of concentration index using generalised linear model: Analysis of socio-economic determinants of health inequality in the northern territory of Australia. Reg Sect Econ Stud. 2013;13(1):183–94.

[23] Yiengprugsawan V, Lim LL, Carmichael GA, Sidorenko A, Sleigh AC. Measuring and decomposing inequity in self-reported morbidity and self-assessed health in Thailand. Int J Equity Health. 2007;6:23. doi: 10.1186/1475-9276-6-23 18088434 PMC2242789

[24] Darmon N, Drewnowski A. Does social class predict diet quality? Am J Clin Nutr. 2008;87(5):1107–17. doi: 10.1093/ajcn/87.5.1107 18469226

[25] Darmon N, Drewnowski A. Contribution of food prices and diet cost to socioeconomic disparities in diet quality and health: A systematic review and analysis. Nutr Rev. 2015;73(10):643–60. doi: 10.1093/nutrit/nuv027 26307238 PMC4586446

[26] RezaeeDaryakenari B, Asadzade P, Thies CG. Economic sanctions and food consumption: Evidence from Iranian households. Int Stud Q. 2024;68(3). doi: 10.1093/isq/sqae103

[27] Kirbiš A, Branilović S. Education and meat consumption and reduction: The mediating role of climate literacy. Foods. 2025;14(19):3333. doi: 10.3390/foods14193333 41097503 PMC12523753

[28] Kirbiš A, Korže V, Lubej M. Predictors of meat reduction: The case of Slovenia. Foods. 2024;13(15):2346. doi: 10.3390/foods13152346 PMC11311667 39123537

[29] Backholer K, Spencer E, Gearon E, Magliano DJ, McNaughton SA, Shaw JE, et al. The association between socio-economic position and diet quality in Australian adults. Public Health Nutr. 2016;19(3):477–85. doi: 10.1017/S1368980015001470 25989940 PMC10271031

[30] Hashimoto A, Murakami K, Kobayashi S, Suga H, Sasaki S, Three-generation Study of Women on Diets and Health Study Group. Associations of education with overall diet quality are explained by different food groups in middle-aged and old Japanese women. J Epidemiol. 2020;31:280–6. doi: 10.2188/jea.JE20200030 32418938 PMC7940974

[31] Koç H, van Kippersluis H. Thought for food: Nutritional information and educational disparities in diet. J Hum Cap. 2017;11(4):508–52. doi: 10.1086/694571

[32] Carrasco-Marín F, Parra-Soto S, Bonpoor J, Phillips N, Talebi A, Petermann-Rocha F, et al. Adherence to dietary recommendations by socioeconomic status in the United Kingdom biobank cohort study. Front Nutr. 2024;11:1349538. doi: 10.3389/fnut.2024.1349538 38751735 PMC11094746

[33] Galobardes B, Morabia A, Bernstein MS. Diet and socioeconomic position: Does the use of different indicators matter? Int J Epidemiol. 2001;30(2):334–40. doi: 10.1093/ije/30.2.334 11369739

[34] Miller V, Webb P, Cudhea F, Shi P, Zhang J, Reedy J, et al. Global dietary quality in 185 countries from 1990 to 2018 show wide differences by nation, age, education, and urbanicity. Nat Food. 2022;3(9):694–702. doi: 10.1038/s43016-022-00594-9 37118151 PMC10277807

[35] Brandt E, Levin A, Holland M, Ferrucci L. Demographic, lifestyle, and dietary factors associated with red meat consumption among a cross-section of New York City residents. IJDRP. 2021;3(2). doi: 10.22230/ijdrp.2021v3n2a301

[36] Hopwood CJ, Zizer JN, Nissen AT, Dillard C, Thompkins AM, Graça J, et al. Paradoxical gender effects in meat consumption across cultures. Sci Rep. 2024;14(1):13033. doi: 10.1038/s41598-024-62511-3 38871837 PMC11176159

[37] Lehto E, Kaartinen N, Sääksjärvi K, Männistö S, Jallinoja P. Background characteristics of meat consumption exceeding the Finnish Dietary Guidelines in 2000-2017. Eur J Public Health. 2019;29(Supplement_4). doi: 10.1093/eurpub/ckz186.282

[38] Liobikienė G, Brizga J. Determinants of meat consumption: Applying the expanded Theory of Planned Behaviour in Latvia. Clean Respons Consum. 2025;16:100247. doi: 10.1016/j.clrc.2024.100247

[39] Barnett K, Mercer SW, Norbury M, Watt G, Wyke S, Guthrie B. Epidemiology of multimorbidity and implications for health care, research, and medical education: a cross-sectional study. The Lancet. 2012;380(9836):37–43. 10.1016/S0140-6736(12)60240-2 22579043

[40] Harris J, Battersby J, Gordon J, Isaacs A, Ranta R, Recine E, et al. Equity and equality in diets and nutrition: Frameworks, evidence, and four country case studies. Food Sec. 2025;17(4):1023–37. doi: 10.1007/s12571-025-01537-5

[41] Odoms-Young A, Brown AGM, Agurs-Collins T, Glanz K. Food insecurity, neighborhood food environment, and health disparities: State of the science, research gaps and opportunities. Am J Clin Nutr. 2024;119(3):850–61. 10.1016/j.ajcnut.2023.12.019 PMC10972712 38160801

[42] Valli C, Maraj M, Prokop-Dorner A, Kaloteraki C, Steiner C, Rabassa M, et al. People’s Values and preferences about meat consumption in view of the potential environmental impacts of meat: A mixed-methods systematic review. Int J Environ Res Public Health. 2022;20(1):286. doi: 10.3390/ijerph20010286 36612609 PMC9819158

